# “*There Was Some Kind of Energy Coming into My Heart*”: Creating Safe Spaces for Sri Lankan Women and Girls to Enjoy the Wellbeing Benefits of the Ocean

**DOI:** 10.3390/ijerph19063342

**Published:** 2022-03-11

**Authors:** Martina Burtscher, Easkey Britton

**Affiliations:** 1 SeaSisters Sri Lanka, Weligama 81700, Sri Lanka; 2 European Centre for Environment and Human Health, University of Exeter Medical School, Knowledge Spa, Truro TR1 SHD, UK; hello@easkeybritton.com

**Keywords:** surfing, surf therapy, gender, sport for development, qualitative, Sri Lanka

## Abstract

Worldwide, there is growing recognition of the wellbeing benefits of accessing and engaging with healthy blue spaces, especially seas, coasts, and beaches. However, vast gender inequalities persist that impact women’s and girls’ ability to safely access these spaces for recreational benefit. This is even more pronounced in the context of emerging surf cultures in regions such as Southeast Asia. Using a qualitative and reflective approach, this paper explored how safe spaces for female surfers are created, using case studies from two female-focused surfing programs in Sri Lanka. To facilitate a safe space, the multi-layered challenges that female surfers face were analysed. The common mediators that enable females to participate in surfing were then investigated and identified, including: seeing surfing as an option, supportive families and communities, the group factor, free lessons, an all-female environment, culturally appropriate surf apparel, and a safe and playful methodology. This study highlights pathways for how unsafe spaces of exclusion and fear may be transformed into safe spaces of inclusion, healing, and empowerment. These findings have implications for how safe spaces may be facilitated for other organisations, as well as the sustainability of female access to surfing, beyond the life of surfing programs.

## 1. Introduction

The past decade has seen a resurgence in women’s and girls’ interest in participating in surfing, along with measures to reduce gender inequalities in the sport, such as pay parity for professional surfers. Recent trends show increasing participation of female surfers from diverse ethnic backgrounds and social classes, from low-income countries with emerging surf cultures [1], especially in regions of Southeast Asia such as the Maldives, Philippines, India, and Sri Lanka. In response, there has been a rapid increase in surfing and development organisations (SDOs) and programs specifically for women and girls in the last five to ten years. Surf scholarship for female surfing is also on the rise. However, this research is largely focused on the Global North, within established surf cultures, and is mostly concerned with female representation [2,3]. With a few notable exceptions, very few studies have addressed the experiences of female surfing in the Global South within emerging surf cultures [4,5,6,7]. Furthermore, as feminist surf scholars have noted, coasts, beaches, and surfing spaces are constrained by numerous injustices and can be highly exclusionary, especially for female bodies [8,9,10,11].

Globally, the relationship between women and the sea has long been underrepresented and poorly understood. Vast gender inequalities persist that impact women’s and girls’ ability to safely access seas, coasts, and beaches, especially for recreational and health benefits [12]. Studies have shown that women disproportionately suffer the impacts of disasters, severe weather events, and climate change because of cultural norms and the inequitable distribution of roles, resources, and power [13]. In the wake of the 2004 tsunami, an Oxfam report found that surviving men outnumbered women by almost 3:1 in Sri Lanka, Indonesia, and India. These cultural norms limit women’s ability to access lifesaving skills such as swimming. There are enormous gender gaps in the ability to swim unassisted, with 85% of women in low-income countries unable to swim [12]. All of this contributes to establishing the ocean as a dangerous, risky, and unsafe space for women and girls.

This paper addresses these gaps and issues by examining the personal experiences and stories of pioneering female surfers within the very masculinised emerging surf culture of Sri Lanka. The paper explores how female surfers and SDOs are creating gendered “safe spaces” of inclusion within surfing. The authors investigated the dynamic interplay of relationships, space, gender, cultural norms, and class in how these safe spaces are established, including the importance of developing processes that build local leadership and promote long-term viability and legitimacy. The authors advocated an intersectional approach as a research framework, “that engages with differences between groups and between individuals and the dynamic process of marginalisation” [2] (p. 148).

There are a growing number of studies that evidence the positive health and wellbeing benefits of access to “blue spaces” [14,15], especially surfing and surf therapy programs [16,17]. Various mediators or enabling factors have been identified for realising these health and wellbeing outcomes, among them the creation of safe spaces and social connection [18]. In addition to these mental health benefits, surfing can also contribute to empowerment, environmental awareness, and economic benefits [5,8,19]. However, what is overlooked in these studies is how to facilitate a safe space, alongside the potential variance of safe spaces for different populations, especially for women and girls. This paper seeks to address this research gap, by exploring how safe spaces are facilitated for women and girls in the context of Sri Lanka.

### 1.1. Safe Spaces for Females in the Sport-for-Development Context

In the sport-for-development (SFD) discourse, the concept of safe spaces is still under-theorized and under-researched, even though it is “critical both to the provision of inclusive and equitable sport opportunities and to leveraging the positive social impacts that can flow from these opportunities” [20] (p. 634). Brady [21], in the context of young women in sport in the Global South, suggested that a safe space for females is not only constituted by its physical setting, but more importantly, by its emotional and psychological dimension. According to Brady, a safe space is free from physical and emotional threat, private and confidential, not subject to intrusions by males and unwanted authority figures, conveniently located and familiar to program participants, and culturally acceptable to parents and other gatekeepers, yet free from parental pressures. Brady also highlighted that creating a safe space is not only about eliminating threats, but also about facilitating respect and dignity. In a safe space, women and girls can develop valued life skills, form new friendships and social networks, enjoy freedom of expression and movement, receive mentoring support, and benefit from new opportunities [21]. Based on these insights, a safe space has a double meaning: it can help facilitate access for females to participate in SFD programs, while it can also contribute to positive outcomes such as women’s empowerment. This study highlights not only the benefits of facilitating safe spaces for female participants, but how doing so has the potential to challenge limiting and harmful gender perceptions and biases.

### 1.2. Women’s Surfing and Gender Equity in Sri Lanka

Sri Lanka is a top surfing destination in Asia, known for its mellow waves, which attract surfers from all over the world. The surf season on the south and west coast occurs from December to March and on the east coast (including Arugam Bay) from May to October. Local male surfers have been surfing since the 1960s [22]; however, local female surfing is a very recent development. From 2015 to 2021, women’s surfing has faced a strong push, supported by SDOs such as *Girls Make Waves (GMW)* and the *Arugam Bay Girls Surf Club (ABGSC)* on the east coast and the *Kids Surf Club Meddawatta* and *SeaSisters* on the south coast, with a few dozen females surfing in 2021. Whereas women and girls from rural areas tend to participate in surfing programs by SDOs, female surfers who grew up in the capital city or abroad usually surf “independently” [5]. Most are beginner surfers, while a few are already competing at the national level and training to become surf instructors. Despite these developments, surfing is still a male domain, and for many females, surfing is still not an option. Most women and girls cannot swim, and they often face socio-cultural, economic, and institutional barriers to participate in surfing. These barriers are the result of the underlying power structures in which surfing takes place [5].

In Sri Lanka, society is based on a patriarchal system [23]. Although Sri Lankan women gained the right to vote in 1931 and have free access to state health services and education, they are under-represented in politics, the labour force, and sport [24,25]. In the private sphere, women are often restricted in decision-making, which is considered a male domain. This all contributes to the continuing subordination of Sri Lankan women. In this regard, it is important to consider intersectionality. For example, women from conservative families in rural areas tend to be less independent in their decision-making and mobility than urban women from middle-class backgrounds [23]. Furthermore, at each stage of a woman’s life cycle, from childhood to puberty to adulthood and, for some, motherhood, there are different socio-cultural norms that must be negotiated.

In the context of surfing, in particular gender norms, societal expectations, and traditions have prevented females from entering the sport [5]. Women and girls from rural areas are usually expected to stay at home and care for their families, rather than engaging in recreational activities in the public space. When girls reach puberty, they tend to become more shielded from society and their interactions with boys are limited by their families [23]. In Sri Lanka, surfing is often associated with the so-called beach boy culture of parties, sex, and alcohol [26]. This can create tension within households, fearful that their daughters might get a bad reputation [5]. Furthermore, for females with lower socio-economic backgrounds, surf equipment and lessons are often not affordable. Poverty and, consequently, prioritizing survival needs are a primary barrier that prevents Sri Lankan women from participating in sports in general [25]. At an institutional level, surfing as a sport is slowly becoming more professionalized; however, most positions in governing bodies, such as the *Surfing Federation of Sri Lanka*, are occupied by men, and there is a lack of female surf instructors from Sri Lanka [5].

On top of these barriers, surfing happens in an element that is often feared. Sri Lanka has one of the highest drowning rates in the world [27], and most people grow up disconnected from the ocean, perceiving it as a place of extraction rather than of recreation [28]. The ocean is regarded as a dangerous place; this internalized fear partly stems from the tsunami that hit Sri Lanka on the 26 December 2004 and killed more than 31,000 people in the country [29]. Furthermore, the beaches and the sea are a males’ domain, who often work as fishermen, surf coaches, lifeguards, or in the navy.

To sum up, surfing is not considered a safe activity for Sri Lankan women and girls for reasons that are often linked to socio-cultural expectations and to the dangers of the ocean. Consequently, many Sri Lankan families do not allow their daughters or wives to participate as they want to protect them. Nevertheless, the situation is slowly changing, and women’s surfing is on the rise.

### 1.3. Study Aim

This paper explores *how* safe spaces for female surfers are facilitated, illustrated by the case studies of the Sri Lankan surfing programs *GMW* and *SeaSisters*. Specifically, the following research questions are investigated:

- What factors enable females to participate in surfing programs?
- Which elements constitute a safe space?

By examining the complexities, inequalities, and possibilities for Sri Lankan females to access and experience the sea, the study’s goal is to show pathways for how unsafe spaces of exclusion and fear may be transformed into safe spaces of inclusion, healing, and empowerment.

## 2. Materials and Methods

### 2.1. Study Settings and Surfing Programs

This study drew on the experiences of female surfers from two surfing programs the lead author has been involved with: *Girls Make Waves (GMW)* and *SeaSisters*. *GMW* is a female-focused surfing program in Arugam Bay on Sri Lanka’s east coast, founded in July 2015 by Tiffany Carothers, an American expat working for the organisation *Surfing the Nations (STN).* Participants are from the Sinhalese–Tamil community in Arugam Bay (which is predominantly a Muslim area) and are between 10 and 44 years old. Surf lessons usually take place once a week during surf season (May–October) led by female international volunteers from *STN*. The program occasionally integrates other activities such as swimming, volleyball, yoga, or self-defence classes. The program has been running from 2015, with occasional lulls due to resistance from community leaders in 2016–2017 and the COVID-19 pandemic since 2020. As part of her Master’s thesis, the lead author spent three months performing field research at *GMW* in Arugam Bay, from July until October 2017. Participants of GMW also formed the *Arugam Bay Girls Surf Club (ABGSC)*, the country’s first all-female surf club, in August 2018. Initially, the club was facilitated by international surfers, including the lead author, and today, it is run by local women for local women.

*SeaSisters* is a female-focused social business with a swimming and surfing program in Weligama on Sri Lanka’s south coast, founded in November 2018 by Amanda Prifti and the lead author. Participants are largely from the Buddhist–Sinhalese community, aged 8 to 52 years old. Lessons usually take place once a week during surf season (November–April) and are divided into sessions at the beach and the swimming pool supervised by a team of international female swim and surf instructors and assistants. Educational components around gender, ocean safety, and environmental awareness are integrated into the lessons. The program has been running for two seasons (2018–2019 and 2019–2020) and, after a two-year pause due to the COVID-19 pandemic, is back operating again.

### 2.2. Study Design and Data Collection

This study was designed retrospectively, drawing on a rich body of experiences and data that the lead author had collected in Sri Lanka between 2017 and 2020, while performing research for her Master’s thesis “Women Making Waves” [5] and co-facilitating surfing programs. The study, therefore, adopted an ethno-case study methodological framework combining ethnography practices with case study research, which allows for sensitivity and valuable insights into how female surfers experience the sea and surf [30,31]. Combining the following primary and secondary data sources helped give “voices to participants” and their interpretation of reality [32] (p. 219), creating a “thick description” of the experience of safe spaces [33] (p. 10):

- Semi-structured interviews with 18 female surfers from the lead author’s Master’s thesis in 2017;
- Two expert interviews with founders of *GMW* and *Kids Surf Club Meddawatta* from the lead author’s Master’s thesis in 2017;
- Participatory observations during 40 swimming and surfing sessions (at *GMW,* the *ABGSC*, and *SeaSisters*) between 2017 and 2020;
- Participant evaluations of the *SeaSisters* program from 2019;
- Informal conversations with female participants between 2017 and 2020 (at *GMW,* the *ABGSC*, and *SeaSisters*);
- Two follow-up interviews with *SeaSisters* participants in 2020;
- Review and analysis of online videos documenting Sri Lankan female surfing (an overview of the videos is provided in Appendix A)

As part of the lead author’s Master’s thesis [5], 18 women were interviewed, sharing insights on their personal experiences of being a female surfer from Sri Lanka. Interviewees ranged from beginners to intermediate, aged 16 to 44 years old. Most of them learned how to surf through *GMW* and grew up in working-class families in the Sinhalese–Tamil community in Arugam Bay, identifying as Christians, Buddhists, and Hindus. In contrast, four interviewees surfed independently outside of *GMW*, who grew up in the capital or abroad and shared a more privileged background. Interviewees were recruited through *GMW*, as well as through posting requests in Facebook groups and asking people involved in the surf scene. To obtain the women’s personal stories, semi-structured qualitative interviews supported by an interview guide were carried out. Interview topics included asking the women about their experiences as female surfers, access to surfing, the benefits and challenges of surfing, as well as their relationship with the ocean. The interviews lasted between one and three hours and were either conducted in English or in Sinhala with the help of a translator. All interviews were recorded and transcribed. In addition, two expert interviews with the founders of *GMW* and the *Kids Surf Club Meddawatta* were conducted, who shared insights on how they facilitate their programs.

This paper also drew on the lead author’s participatory experiences delivering *GMW,* the *ABGSC*, and particularly, *SeaSisters* lessons from observations and informal conversations with participants, considering organisational mediators and practices, rather than the personal perspectives of the female surfers, per se. At *GMW* lessons in 2017, a researcher’s journal was kept. As part of the *SeaSisters* program’s evaluation in 2019, seven participants (aged 9 to 44 years old) were interviewed about their experiences with the swim and surf lessons. In this study, pseudonyms were used for all interviewees and participants, with the exception of public sources and interviewees who requested their real names be cited.

### 2.3. Data Analysis

Following a review of the relevant literature to investigate the barriers female surfers face in the Global South, and specifically Sri Lanka, the ocean as an “unsafe” space emerged as a key theme (as outlined in the previous section). Building on the lead author’s in-depth, practitioner-led experience with female surfers in Sri Lanka, existing data were revisited to examine this theme and what mediators might be for transforming the sea and surf into a safe and enabling space. Primary and secondary data sources were analysed using an inductive, grounded theory approach [34]. Each author individually coded the data to identify emerging concepts first through open coding, before forming subcategories. Relationships between these categories were identified with core themes emerging. To avoid possible bias, constant comparison between data sources and the literature was carried out to check for consistency. This approach to data analysis was adopted because of its flexibility to integrate emerging concepts around the notion of safe spaces for female surfers in Sri Lanka.

Overall, the perspectives in this study reflect a broad mix of ages from 8 to 52 years old and a mix of ethnicity, socio-economic classes, religions, educational backgrounds, and surfing abilities. When analysing the insights, the lens of intersectional feminism played an important role, as the female surfers’ experiences varied not only on gender, but also on other factors, such as age, class, or regionality.

### 2.4. Researcher Position

The authors acknowledge that they hold insider and outsider perspectives as both scholars and surfers. As white female scholars of European descent, they are privileged outsiders performing research about “other” women from the Global South. To avoid othering processes, the authors constantly tried to reflect upon their positions and hierarchies, considering insights from post-colonial scholars, such as Spivak [35] and Mohanty [36]. As surfers and practitioners, both authors are also active participants in surfing; however, the lead author’s long-term involvement in the co-production of female surfing spaces in Sri Lanka offers a unique and challenging insider perspective.

The lead author was immersed in the case study areas from 2017 to 2020, staying in Sri Lanka for up to two years at a time. She also participated actively as an instructor/supervisor in over 40 surf lessons at *GMW*, the *ABGSC*, and *SeaSisters* and was involved in program design and delivery. It is important that the lead author’s multi-dimensional role as a researcher from the Global North, a surfer, a practitioner, and a friend of the interviewees be critically reflected on [37]. The lead author’s position as a female surfer facilitated a greater sense of familiarity and connection with other female surfers. The longer-term nature of the study and direct involvement in the programs permitted a shift from “outsider” to “insider” status for the researcher, revealing intersectional nuances and differences that may otherwise have remained invisible. Despite this shared bond and insight, through critical self-reflection, she constantly tried to challenge her eurocentrism. The authors acknowledge that they cannot guarantee the possible misinterpretation of certain phenomena. This research process emphasises that the researchers were also learning from the participants, who challenged and enriched the researchers’ perspectives on what it means to be a woman. As surfers, the authors also acknowledge that every woman and girl experiences surfing differently, and what might be empowering for some might be even disempowering for others. In what follows, the authors sought to integrate the voices of the female surfers and allow their stories to speak for themselves.

## 3. Results and Discussion

**Figure 1:**
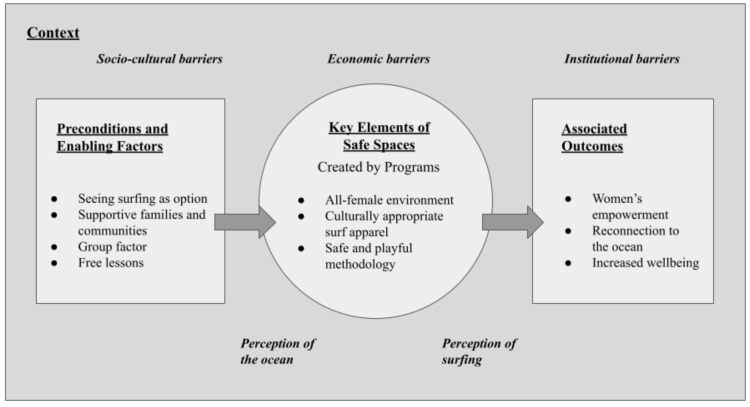
Logic model showing the mechanisms of creating a safe space for females in Sri Lanka.

This section explores the complexities, inequalities, and possibilities for Sri Lankan women and girls to access surfing and illustrates how safe spaces can be facilitated by surfing programs. In the first step, the context and barriers are analysed. Based on these insights, mediators (preconditions, enabling factors, and key elements) for facilitating a safe space are identified and discussed. Finally, the implications and limitations of the findings are outlined. Figure 1 summarises the results in a logic model that was inspired by the surf therapy program theory developed by Marshall et al. [16], adapting it to the setting of facilitating a safe space for females while considering the local context.

As illustrated in Figure 1, surfing does not happen in an isolated space, but is embedded in underlying social structures and power dynamics. The context shapes the constitution of a safe space. Furthermore, it is important to acknowledge that only when certain preconditions are met, women and girls can access the safe space. In turn, learning how to surf in a safe space can lead to associated outcomes such as women’s empowerment and reconnecting to the ocean. In the following, all elements shown in Figure 1 are analysed separately.

### 3.1. Context and Implications for Program Design

Facilitating a safe space for women and girls requires careful planning and managing [21,38]. When designing a program, it must be analysed *whom* the program wants to target, *what* constraints they are facing, and *how* these barriers can be lowered specifically for them. It is necessary to understand the context in which surfing takes place and to identify the multi-layered barriers that women and girls are facing. As highlighted in the Introduction, various socio-cultural, economic, and institutional constraints can hinder Sri Lankan females from participating in surfing. In this regard, an intersectional lens is essential. Not every Sri Lankan female faces the same challenges; barriers can vary individually and are influenced by categories such as class, ethnicity, family background, religion, education, or location. In particular, there are significant differences in barriers for female Sri Lankan surfers who grew up in the capital or abroad and women from rural villages with a lower socio-economic background. Barriers are also fluid and can change during their lives. For example, when girls turn into “big girls” after having their first menstruation, they often face more restrictions in participating in public life than before. In contrast to men, women typically assume domestic duties and care-taking roles within households, with additional constraints on their time when they become mothers. Unpaid domestic work is often in addition to their paid work outside the home [23]. Carrying this double burden, it can be challenging for women to find time for surfing.

Table 1 presents the barriers and mediators identified for the surfing programs run by *GMW* and *SeaSisters*. *GMW* and *SeaSisters* did not use this particular method when designing their programs; this analysis was performed retrospectively in the research process.

In the following sections, the preconditions, enabling factors, and key elements presented in Figure 1 are discussed in detail, as well as the role of each mediator (Table 1) and how these factors can enable a safe space.

### 3.2. Preconditions and Enabling Factors

#### 3.2.1. Seeing Surfing as an Option

Before participating in surfing or in a surfing program, women and girls first have to realize that surfing is even an option for them. As *SeaSisters* participant Sanu put it: “I grew up by the ocean. But I have never seen it as a place for me. They say, Sri Lankan girls don’t surf. It’s hard to believe what you don’t see” (Sanu, video WRLW 2021). As her, many female surfers, both from the east and south coast, report that they never thought it would be possible for them to surf. In part, this is related to the fact that they have never seen local female surfers when growing up. As Baby Rani, a surfer from Arugam Bay, stated: “My father surfs. My brother surfs. My husband surfs. When we go to the beach, we can only watch boys surf” (Baby Rani, video Girls Make Waves 2019). Furthermore, Mahishaa, a former competitive swimmer and *SeaSisters* volunteer, explained that even though she had learned how to swim in the ocean at an early age, it never occurred to her that she could surf as well. In this regard, external interventions and local role models can show new possibilities [39].

In the biographies of most Sri Lankan female surfers, an external push played an important role. Many women only tried surfing because they were encouraged by others, either by friends, tourists, family members, or surfing programs. In particular, the latter can open up a space that did not exist before. For example, Ayomi from Arugam Bay was interested in surfing, but she thought she could not do it alone. When *GMW* started, she and other girls had the chance to learn it together in a group. As Susanthika recalled: “When I saw everyone was surfing, it made me go for it” (Susanthika, interview in 2017). Similar observations were made at *SeaSisters*. The word quickly spread among the villages, and many women and girls reached out that they wanted to learn how to swim and surf as well. Eventually, the program could not keep up with the demand anymore and had to introduce a wait list. These examples show that there is a real interest among Sri Lankan females to learn how to swim and surf, once they view it as a possibility. This, in turn, can be linked to the concept of women’s empowerment and a “shift in consciousness” [40] (p. 3), where women reshape their imagination of what is possible for them to be and to do [40,41].

Furthermore, local role models can be “catalysts for change” [42] (p. 126), by showing a culturally relevant, new type of surfer and influencing women’s self-perceptions [39]. For example, Mandari, a free surfer from the capital, reported that she initially felt inspired by Indian surfer Ishita Malaviya, a woman to whom she could relate to more than to Western surfers, as they share similar circumstances under which they learned how to surf, “[…] for me it is just, ‘Wow, if she can do it. Why can’t I?’” (Mandari, interview in 2017)

Furthermore, Shamali Sanjaya, the first female surfer from Arugam Bay, has become a role model in her community and beyond. Initially, she faced backlash for contesting gender norms, but over time, she proved that there is “nothing bad” about surfing and that she surfs while keeping her cultural values. Today, she has earned more respect for her efforts to take part in a sport which is considered a male domain, paving the way for aspiring female surfers to follow. Her story is particularly relevant for women and girls from rural areas, who can relate to her as they grew up in a similar context.

The power of local role models also becomes very visible when connecting the stories of *GMW* and *SeaSisters*. When participants of *GMW* established the *ABGSC*, their accomplishment was widely shared on social media and local newspapers, which was eventually the springboard for *SeaSisters* to start an initiative on the south coast. As evident from these stories, the ability of female surfers to share their experiences and exchange knowledge helps to highlight alternative possibilities that challenge existing gender inequalities.

#### 3.2.2. Supportive Families

Another factor identified as a precondition for Sri Lankan females to participate in surfing is the support of their families. At *GMW* and *SeaSisters*, all participants have their families’ permission. Similar to Brady, it is argued that even when the women and girls have interest, it can still remain challenging for them to participate because of familial concerns [21]. Particularly in rural areas, it is common that women and girls have to ask their families, especially their male family members or husbands, for permission to participate in activities outside the household [23]. Thus, it is vital to understand their perspectives, to build trust, and to facilitate a space that is considered as safe and culturally acceptable by both participants and their families [21].

For many families from rural areas, it is important that young women of the household have a good reputation. For example, Anju, a male surf instructor from Weligama, was initially worried about his sister becoming engaged in surfing: “I didn’t think Sanu should surf. In Sri Lanka, the culture believes girls who surf will end up in the wrong crowd and damage their reputation. I believed in this mindset too” (Anju, video WRLW 2021). Their father Kumara is supportive of his surfing daughter, but reported that his friends did not understand it: “Even my friends say that she might lose her beauty because of surfing, and boys will not be interested in her then” (Kumara, Sri Lanka, video WRLW 2021). This statement reflects the widespread concern that young women will not find a husband when they go against the norm and behave in ways that are considered “untypical” for females by society. Other families are more afraid that surfing in the ocean is a dangerous activity, as Shamali reported: “My brother was not happy with me surfing. He felt it was too dangerous with the big waves that are at the sea” (Shamali, Sri Lanka, video Roar Media 2019). Mahishaa, who grew up in Colombo, explained that her parents did not take her surfing seriously: “I face challenges when my parents and my family consider surfing as something to distract me, something that they think I waste my time on” (Mahishaa, Sri Lanka, interview in 2020).

Consequently, SDOs need to build trustful relationships with families and show them that surfing is something beneficial for their daughters, sisters, or wives, providing various physical and mental benefits [15,16] and economic opportunities [5]. The case studies of the female surfers also illustrate that some family members may change their opinions over time. For example, pioneering surfers Shamali and Sanu were not supported by their brothers in the beginning. However, when they improved their skills and surfing became more common for females, they even became proud of their sisters: “Now I see surfing is not bad for girls. Every good change that comes, comes from somebody being brave and doing something new” (Anju, Sri Lanka, video WRLW 2021).

Additionally, it needs to be considered that even when families give their permission, it might be challenging for women to find time for surfing, as they are usually responsible for household duties and childcare. In particular, mothers rely on supportive husbands, family members, or friends to look after their children while they are in the water. In this regard, couples such as Shamali and her husband, who is very supportive of her surfing, show new possibilities and might influence traditional gender roles.

#### 3.2.3. Supportive Communities

Besides the crucial support of their families, it is also important that local communities are supportive. In Sri Lanka, female surfing is perceived as controversial, and women may face disempowering consequences as they increasingly enter the public space and contest gender norms [5]. Most female surfers from Arugam Bay reported that they have faced negative reactions at least once. Questioning traditional gender roles can lead to intolerance, irritation, and resistance from community members [43]. For example, in Arugam Bay, the organisers and participants of *GMW* faced backlash from some male community members in their early days. It was reported that they were afraid of a cultural change, that the surfer girls would start to “dress like the women from Colombo”. Eventually, the resistance became so strong that some families took their daughters out of the lessons and the program had to pause. Also other studies in the SFD context reported potential negative consequences [44,45]. In contrast, *SeaSisters* as an organisation has not faced any resistance by local community members; however, there is also a lack of data on how the program has been perceived by local communities. *SeaSisters* participants come from several spread-out villages along the south coast, perhaps allowing greater freedom from being under observation by members of their community, unlike their counterparts in Arugam Bay, who all come from and surf in the same village and are constantly observed. That said, further research on the perception of their communities would need to be conducted.

The case study of the female surfers from Arugam Bay also shows that opponents of female surfing may change their mindsets over time. For instance, the head of the local tourism board initially told the organisers of *GMW* to “buy them sewing machines” instead of taking them surfing. However, when the tourism board realized there was an economic opportunity in local female surf instructors giving lessons to female tourists, they started to actively support the women in pursuing their surfing careers. This example illustrates that reshaping men’s perception of appropriate roles for females is crucial, as their behaviour in the public and private sphere influences women’s and girls’ mobility and their participation in public life [21]. Similarly, Rowlands argued that for women’s empowerment to be substantial and sustainable, it is especially important that the attitudes and the behaviour of men change [41]. These findings highlight the relevance of male support for enabling women’s participation in surfing.

#### 3.2.4. Group Factor

Another enabling factor for participating in a surfing program is that females surf together as a group. As Ayomi stated: “We can’t go alone. We can go with friends. But not alone” (Ayomi, interview in 2017). She and other young women from Arugam Bay reported that they are not allowed to walk alone in the public space. They also do not feel comfortable going to the beach by themselves, as local male visitors would make comments to them. In turn, if people saw them talking to a man, even just casually, they might spread rumours about them. According to Brady, restricting young women’s mobility when reaching puberty is a common phenomenon in countries in the Global South, where “adolescence is a time when the world expands for boys and contracts for girls” [21] (p. 39). Consequently, going surfing with others is the only way that some women and girls can access surfing. On the south coast, one *SeaSisters* volunteer reported that she asked the grandfather of a young girl many times to take her surfing, but was always denied. However, when *SeaSisters* started, he finally allowed his granddaughter to surf as he saw that other girls from the community were doing it. It is argued that in a group, it is not a single girl or woman standing out of the crowd and going against the norms that might make community members less likely to say something against them. Susanthika explained:

“*Yeah, it will get better when like all the girls are bold and just go surfing and say, ‘We want to do this.’ It will start to change then. And then, people have no other choice than accepting it. […] the family will not talk about other girls because they have already one girl in their family who surfs*.”

Susanthika (21 years), interview in 2017.

The “group factor” can also lead to confidence and collective power for women [41]. Particularly at *GMW,* the women feel part of a movement through their shared identity. They want to fight for their rights, as Shamali said: “It (surfing) is not just for men. As girls in this country, we don’t have to keep our heads down. We need to show that we are equal to them. We need to show that to the world” (Shamali, Sri Lanka, video Roar Media 2019). Consequently, surfing in a group does not only enable women′s participation, but also constitutes a sense of belonging and sisterhood [46]. This process is closely linked with women’s empowerment on a collective level, where women realize that they can have a bigger influence together than alone and use their “power with” to negotiate their interests [41].

#### 3.2.5. Free Lessons

According to Nanayakkara, the prioritization of survival needs is one of the main reasons why Sri Lankan women are generally more excluded from sport [25]. Similarly, *SeaSisters* participant Kalpa thinks that for many women, priorities lie on earning money rather than on recreation. This topic, however, is not only related to gender, but also a matter of class. Especially for women from poor rural areas, surf equipment and lessons are often not affordable. To overcome this economic barrier, both *GMW* and *SeaSisters* offer their lessons for free.

### 3.3. Key Elements of a Safe Space

As stated in the Introduction, a safe space includes a physical and emotional dimension and is not only characterised by eliminating risks and threats, but also by facilitating a positive and empowering experience. Surfing needs to take place in a way that is considered safe and culturally acceptable both by participants and their families. In the context of *GMW* and *SeaSisters*, the following elements have been identified as key elements: an all-female environment, culturally appropriate surf apparel, and a safe and playful teaching methodology.

#### 3.3.1. All-Female Environment

In the SFD context, women and girls can either participate in mixed-gender or female-only sport programs, whereas both approaches have benefits and pitfalls. The “right” approach depends on the specific context, structural restrictions, and realities of the targeted women and girls [47,48,49]. The organisation *Women Win* suggests that in some cultures, it is necessary to create a female-only space for adolescent girls to feel physically and emotionally safe [38]. In Sri Lanka, both *GMW* and *SeaSisters* create an all-female environment, which counts for participants and instructors. It is argued that in their context, a female-only space can contribute to a higher level of physical and emotional safety, in particular for young women, and that parents are more likely to give their permission. As children, Sri Lankan boys and girls can usually both play outside and mix with each other. In surfing programs for children at *STN* and the *Kids Surf Club Meddawatta*, boys and girls participate in mixed groups. However, after reaching puberty, Sri Lankan girls are often expected not to mix with boys or men anymore [23]. Kalpa, who also works at SeaSisters as a translator, explained that families want to protect their daughters from “something bad happening to them” and that, “It’s not possible to go to the sea with an unknown man. It’s not comfortable” (Kalpa, informal conversation in 2020). Furthermore, many other participants from *GMW* and *SeaSisters* reported in informal conversations that they feel more comfortable when there are no men around. In addition, being in a female-only space can have empowering effects, such as new support systems, friendships, increased self-esteem, and a sense of belonging [50,51]. For example, *SeaSisters* participants Isuri and Sanuthi reported that they appreciate the fun atmosphere and that everybody supports each other. *SeaSisters*’ lessons also serve as a platform to talk about female-related topics without any judgment. Similar empowering experiences have been reported from female-focused surfing programs all over the world, such as *Like Water* in Iran [4], *Wave Wahines* in England [52], *Brown Girl Surf* in the United States [53], *A Liquid Future* in Indonesia [54] or *Hijas del Mar* in El Salvador [55].

When facilitating surfing programs, it is also crucial to explore the role of coaches and mentors [16]. In the context of surf therapy, Marshall highlighted the importance of “being able to interact and build relationships with adult mentors outside of normal hierarchical structures such as school or family structures” [18] (p. 23) as integral to the associated mental health outcomes. In Sri Lanka, it is a challenge to build a female-only coaching team as there is a lack of qualified *local* female swim and surf instructors. Thus, at *GMW* and *SeaSisters*, foreign women have taken over that role. Considering the North–South context and insights from post-colonial, critical surf, and SFD studies [56,57,58,59], it is important to reflect on the power relations, hierarchies, and dependencies between instructors and participants. At both programs, participants highly appreciate their instructors’ support, and they often build trustful relationships. Instructors also reported that they felt empowered, particularly as they learn more about what it means to be a woman in a cross-cultural setting.

However, the other side to this is that at *GMW* and *SeaSisters*, most participants rely on their international instructors and barely surf outside the facilitated safe space. Most are not yet confident swimmers and surfers, and despite goals to train local women to teach themselves and others, skill building takes time. Consequently, organisations run by foreigners can create dependencies. This became particularly visible when *SeaSisters* stopped their lessons due to the COVID-19 pandemic. Most volunteers returned to their home countries, and participants were not able to continue surfing without their support. In this regard, local leadership is important for long-term success, such as the establishment of all-female surf clubs. *ABGSC* was founded by participants of the *GMW* program who wished to go surfing more regularly. In the beginning, the club was co-initiated and supported by women from the Global North, which then slowly stepped back from their positions. Today, the club is run by local women for local women, which gives ownership and paves the way for the younger generation to follow.

#### 3.3.2. Culturally Appropriate Surf Apparel

Considering that the female body in sport, and particularly in surfing, is a “location for debates about the changing nature of ideology, power, social structures and cultural systems” [60] (p. 1887), it is argued that the choice of sport apparel is another mediator for creating a safe space [6,7]. The women surfers from Arugam Bay and the south coast reported that there is high pressure from Sri Lankan society to look a certain way and that they are expected to dress modestly, both on land and in the water. They highlighted that they also want to dress this way, to “respect their culture” and to “protect themselves” from the male gaze. Furthermore, they are expected to maintain fair skin, a common beauty ideal in South Asia associated with wealth and superiority [61]. For participants at *GMW* and *SeaSisters*, it is important to point out that they want to show new possibilities, while keeping their cultural values. To respect local customs, at *GMW* and *SeaSisters* lessons, all participants and instructors are advised to cover their knees and shoulders, wearing surf leggings and t-shirts, which makes them feel comfortable and free from potential harassment of male observers on the beach. This stands in contrast to the experiences of some female free surfers from urban areas, who tend to surf in bikinis and regularly receive negative comments for it, in particular from local men who consider it inappropriate that they reveal their bodies openly. Some participants at *GMW* and *SeaSisters* expressed that they dislike that woman from Colombo surf in bikinis. These examples illustrate that surfers’ bodies are a particularly “contested terrain” [7] (p. 1170) and that female surfers are constantly negotiating the conformation or contention of socio-cultural gender norms.

#### 3.3.3. Safe and Playful Teaching Methodology

Lastly, high safety standards and a playful methodology are identified as key elements to facilitate a safe space. As highlighted in the Introduction, most Sri Lankan women and girls grow up disconnected from—and sometimes even traumatized by—the ocean and perceive it as a dangerous place [27]. At *GMW* and *SeaSisters,* almost all participants did not know how to swim before joining the programs. Considering this, surfing programs operate in a very sensitive context where the ocean is a constantly changing environment, holding several potential risks such as rip currents. Participants also need to overcome fears or trauma and build essential swimming skills in order to reconnect to the sea as a place that is health-enabling rather than life-threatening. Thus, high safety standards must be provided at all times, including trained staff, translators, and a close ratio between instructors and students. A playful, rather than a technical or performance-oriented, approach has been identified as a key element to promote the wellbeing benefits of the ocean [8,16,31,62]. At *SeaSisters*, a playful teaching methodology was developed, reconnecting the women and girls to the water step by step. When participants start building their swimming skills, they gradually feel more comfortable in the water. At *GMW* and *SeaSisters* lessons, it was often observed that women and girls were very nervous when going surfing for the first time. However, being close with an instructor and staying in the shallow water where they could still touch the ground with their feet made them feel safe. After catching their first waves, participants often got hooked, not wanting to stop anymore. As Sanu reported, “I got the surfing bug. Now I want to surf every day” (Sanu, informal conversation in 2020). Both at *GMW* and *SeaSisters,* most participants reported that they were afraid of waves before learning how to surf—and that through surfing, they overcame their fears. They even started to feel more confident back on land, as Dayani pointed out:

“*Before, I was so afraid. When I started surfing, I learned things which made me so afraid, like standing on a board or paddling. This makes me more confident. This makes me less afraid of things*.”

Dayani (23 years), interview in 2017.

Furthermore, their relationship with the ocean deepened, transforming it from a fearful to a loving one. What is remarkable is that this psychological transformation can even take place in people with trauma. For example, *GMW*-participant Mona from Arugam Bay lost her mother in the tsunami in 2004, which left her deeply traumatised and she had not gone close to the sea for years. When she joined her first *GMW* lesson in 2017 after being convinced by her friends to give it a try, she was extremely nervous. However, she did not give up and managed to ride some waves, lying on her belly. She reported, “There was some kind of energy coming into my heart!” (Mona, interview in 2017) After her first attempt, she kept surfing, wanting to stand up.

“*Surfing makes me forget the tsunami. Now I want to have fun. Before, I was sad about the ocean. Now, I want more. I want to learn more. I want to surf more. Then I also forget my other things [worries] a little bit. Now, my energy is coming like, I want to learn. […] I want to catch my own wave*.”

Mona (31 years), interview in 2017.

Today, Mona goes surfing regularly and even occasionally teaches her two daughters. Furthermore, at *SeaSisters*, similar transformations and positive effects on wellbeing were observed. Sanu, for example, “found herself in the ocean”. Kalpa stated that “*SeaSisters* taught me how much I missed the ocean even though I live near it” (Kalpa, informal conversation in 2020) and that she has started to go on beach walks together with her mother, something they have never done before. These stories are exemplary of the healing power of the ocean, which has also been confirmed in various other studies in the fields of blue space and surf therapy [15,63,64,65].

### 3.4. Outcomes and Limitations

This study focused on how to access and facilitate a safe space, rather than on its associated outcomes. Still, the results suggested that learning how to swim and surf in a safe space can have empowering and health-promoting effects, such as seeing new possibilities, changing cultural perceptions, overcoming fears and trauma, creating new friendships and support systems, learning new skills, and feeling a sense of community, sisterhood, and collective power. The potential of surfing for women’s empowerment was researched in greater detail in a previous study by the lead author, arguing that surfing can contribute to empowerment on many levels. However, surfing can also have disempowering consequences, such as resistance from the local community. Consequently, participants might learn how to surf in a safe space and feel free in the water, but still face challenges back on land. In this study, the potential negative outcomes of surfing programs were only briefly considered and need to be further researched. Of particular interest are the dependencies, ideologies, and hierarchies of surfing programs in a North–South context. Furthermore, the question arises: What are the consequences when surfing programs suddenly stop, such as during the COVID-19 pandemic?

It also must be pointed out that this study has its limitations and blind spots. In Sri Lanka, the challenges and possibilities for females to access the sea are multi-layered, and they are too complex to depict them all in detail in this study. Further barriers and mediators, such as female household duties and the implications for the program’s schedule or the women’s (restricted) mobility to travel to the program and surf spots, could be explored further. The case study was conducted in a very specific context and only considered the perspectives of women who actually participated in surfing programs. However: What are the barriers for women who did not participate? Would they be interested in surfing? What would a safe space look like for them? Even though participants reflected a broad mix of age, location (east and south coast), and ethnicity (Sinhalese and Tamil), other perspectives are missing (e.g., Muslim women, transgender people, women with disabilities). Although the long-term nature of this study did allow for changes in cultural perspectives to be documented, including those of some male surfers, an in-depth analysis of the views of men was beyond the scope of this study. In order to better understand any attitudinal shift in gender bias, the investigation of male attitudes towards female surfers is an important area of further study.

## 4. Conclusions

This study examined how to transform unsafe spaces of exclusion and fear into safe spaces of inclusion, healing, and empowerment, in the context of female-focused surfing programs in Sri Lanka. What makes a safe space cannot be generalised, as it always depends on the individual context and is influenced by existing structures. Surfing, as with any physical activity, is “intertwined with the structures, norms and ideals of a society, and they always mirror that society’s gender order and gender hierarchy” [66] (p. 1). That said, in this case study, common preconditions and key elements that make a safe space were identified. As a starting point to facilitate a safe space, the multi-layered challenges that women and girls face in order to access surfing need to be identified. Particularly in rural areas, Sri Lankan females are confronted with barriers related to gender norms and societal expectations. Furthermore, in Sri Lanka, the ocean is often regarded as a dangerous place, and many women and girls grow up disconnected from the sea, not being able to swim.

Considering the patriarchal system, women and girls can only access surfing when certain preconditions are met, carving out freedoms in a constrained context. Where many females may not see surfing as an option for them, surfing programs (offering access to free group lessons) and local role models can show new possibilities. With women often lacking autonomy in their decision-making, surfing must be executed in a way that is considered as safe and culturally acceptable by participants, their families, and their communities. The necessity to create a safe space is born from the need to overcome these limiting gender norms.

A safe space is not only defined by reducing risks and threats, but also by creating an empowering experience. At *GMW* and *SeaSisters*, the following key elements that constitute a safe space were identified: an all-female environment, culturally appropriate surf apparel, and a safe and playful methodology. A female-only space can increase the emotional and physical safety level for participants; however, also in this context, power relations and dependencies between participants and instructors must be considered. This study highlighted the importance of building local leadership for the long-term viability and legitimacy of the program. If executed in a safe way, surfing can lead to many positive wellbeing outcomes, such as overcoming fears and trauma, reconnecting to the ocean, learning new skills, or creating new friendships and support systems. In addition to these outcomes, the surf programs in this study also have the potential to change negative and debilitating gender perceptions held by male members of society about women.

The importance of understanding the differentiated needs and experiences of female surfers in Sri Lanka, such as those from urban or rural areas, was also highlighted and deserves further research. For female surfers, the desire to experience new possibilities and surf did not necessarily mean a desire to change their culture. As Sanu said, “I don’t want to change our culture. I just want to show girls can do anything boys can do” (Sanu, video WRLW 2021). In this study, the surfing programs contributed to opening up new spaces of possibility, ones that did not exist before. Ultimately, encouraging Sri Lankan women’s participation in surfing “requires a paradigm of changes in the current male dominated sport system as well as to the patriarchal culture” [60] (p. 1900). These findings have important implications for how safe spaces may be facilitated for other organisations, as well as the sustainability of female access to surfing, beyond the life of the programs.

## Figures and Tables

**Table 1:** Barriers and mediators for female participation in surfing in Sri Lanka.

Barriers	Mediators
Do not see surfing as a possibility for them; cannot identify with Western surfer girls	Seeing surfing as an option through external interventions and local role models
Need permission of families to participate in activities outside the home; they have concerns regarding safety and reputation; gendered constraints on time	Support of families through building trust and creating a space that is considered safe and culturally acceptable
Resistance and harassment of local community members and visitors	Supportive communities; changing perceptions of appropriate roles for females
Restricted mobility; expectation not to go surfing alone	Group factor
Prioritization of survival needs; expensive surf lessons and equipment	Free lessons, provision of equipment
Expectation not to mix with men after reaching puberty; women do not feel safe with unknown men	All-female environment (participants and instructors)
Gender norms and expectations around clothing and beauty (modesty, fair skin)	Culturally appropriate surf apparel
Ocean regarded as a dangerous place; women cannot swim; trauma from 2004 tsunami	High safety standards; playful approach; skill-building

## Data Availability

The data presented in this study are available upon request from the corresponding author: martina.burtscher@yahoo.com.

## Appendix A. Links to Videos Documenting Sri Lankan Female Surfing

Bloomberg Media. Making Sri Lankan Beaches a Safe Space. 2020. https://www.bloomberg.com/news/videos/2020-03-06/making-sri-lankan-beaches-a-safe-space-video (accessed on 18 December 2021).

Family Womxn. By Tao Farren-Hefer. 2020. https://www.theinertia.com/surf/this-is-the-story-of-the-first-sri-lankan-womens-surf-club/ (accessed on 18 December 2021).

Girls Make Waves/A-Bay Girls Surf Club. By Robin Mason. 2019. https://vimeo.com/374210705 (accessed on 18 December 2021).

Great Big Story. Sri Lanka’s First All-Female Surf Club. 2019. https://www.youtube.com/watch?v=YW1148iLZH8 (accessed on 18 December 2021).

Roar Media. Girls Make Waves. 2019. https://roar.media/english/life/in-the-know/girls-make-waves (accessed on 18 December 2021).

WRLW (We Are Like Waves). By Jordyn Romero. 2021. https://vimeo.com/478728815 (accessed on 18 December 2021).
